# Surgical treatment of secondary peritonitis: A continuing problem

**DOI:** 10.1007/s00104-015-0121-x

**Published:** 2016-01-08

**Authors:** O. van Ruler, M. A. Boermeester

**Affiliations:** Department of Surgery, Academic Medical Center, 1100 DD P.O. Box 22660, Amsterdam, The Netherlands

**Keywords:** Peritonitis, Abdominal sepsis, Planned relaparotomy, On-demand relaparotomy, Treatment strategy, Peritonitis, Abdominelle Sepsis, Geplante Relaparotomie, Relaparotomie bei Bedarf, Behandlungsstrategie

## Abstract

Secondary peritonitis remains associated with high mortality and morbidity rates. Treatment of secondary peritonitis is challenging even in modern medicine. Surgical intervention for source control remains the cornerstone of treatment, beside adequate antimicrobial therapy and resuscitation. A randomized clinical trial showed that relaparotomy on demand (ROD) after initial emergency surgery is the preferred treatment strategy, irrespective of the severity and extent of peritonitis. The effective and safe use of ROD requires intensive monitoring of the patient in a setting where diagnostic tests and decision making about relaparotomy are guaranteed round the clock. The lack of knowledge on timely and adequate patient selection, together with the lack of use of easy but reliable monitoring tools, seems to hamper full implementation of ROD. The accuracy of the relap decision tool is reasonable for prediction of ongoing peritonitis and selection for computer tomography (CT). The value of CT in an early postoperative phase is unclear. Future research and innovative technologies should focus on the additive value of CT in cases of operated secondary peritonitis and on the further optimization of bedside prediction tools to enhance adequate patient selection for intervention in a multidisciplinary setting.

## Abstract (de)

Eine sekundäre Peritonitis geht immer noch mit hohen Mortalitäts- und Morbiditätsraten einher. Die Behandlung der sekundären Peritonitis stellt selbst in der modernen Medizin eine Herausforderung dar. Neben adäquater antimikrobieller Therapie und Wiederbelebungsmaßnahmen bleibt die chirurgische Intervention zur Therapie der Infektionsquelle eine tragende Säule der Behandlung. Eine randomisierte klinische Studie zeigte, dass die Relaparotomie bei Bedarf ("relaparotomy on demand", ROD) nach initialer Notoperation die bevorzugte Therapiestrategie ist, unabhängig von Schweregrad und Ausmaß der Peritonitis. Der effektive und sichere Einsatz der ROD erfordert eine intensive Überwachung des Patienten in einer Umgebung, in der Untersuchungen zur Diagnostik und die Entscheidung über eine Relaparotomie rund um die Uhr garantiert möglich sind. Fehlende Kenntnisse der zeitgerechten und angemessenen Patientenselektion zusammen mit einem mangelnden Einsatz einfacher, aber verlässlicher Überwachungsinstrumente scheinen die vollständige Etablierung der ROD zu behindern. Die Genauigkeit des Entscheidungsverfahrens für eine Relaparotomie ist von Bedeutung für die Vorhersage einer anhaltenden Peritonitis und die Selektion für eine Computertomographie (CT). Der Wert der CT in der frühen postoperativen Phase ist noch unklar. Zukünftige Forschung und innovative Technologien sollten auf den zusätzlichen Nutzen der CT bei Fällen mit Operation einer sekundären Peritonitis und auf die weitere Optimierung von Verfahren zur Prädiktion am Krankenbett fokussieren, um die adäquate Patientenselektion für eine Intervention in einem multidisziplinären Rahmen zu fördern.

The treatment of secondary peritonitis, or abdominal sepsis, is currently a matter of debate. Mortality and morbidity rates have dropped only slightly during the last decades, even though medical care has markedly improved in developed countries. The origins of secondary peritonitis, the severity, the time span from disease to the onset of treatment, as well as the patients themselves are very heterogeneous. In this overview article we outline the most important aspects of the treatment of secondary peritonitis, with emphasis on the surgical strategy.

## Definitions

Secondary peritonitis is defined as an acute infection of the peritoneum due to loss of integrity of the gastrointestinal tract or other visceral organ. Causes of secondary peritonitis comprise spontaneous perforations (e.g., due to diverticulitis, appendicitis, cholecystitis), traumatic perforation of a visceral organ, or iatrogenic causes (e.g., perforation, anastomotic leakage) [1].

Severe secondary peritonitis, or abdominal sepsis, even in modern days is still characterized by high mortality and morbidity rates due to multiple organ failure (MOF) from septic shock. Reported mortality rates have only decreased slightly over the last few decades, and range from 20 to 60 %. Morbidity rates are as high as 50 % with subsequent long hospital and intensive care unit (ICU) stays [2, 3]. Even though the true incidence of abdominal sepsis is not known, it is regarded as the second most common cause of sepsis [4].

## Initial treatment

### Surgery

The cornerstone of the treatment of secondary peritonitis is prompt elimination of the infectious focus, supported by intensive resuscitation and antimicrobial therapy [3]. Treatment is targeted at source control and prevention of ongoing infection. Prompt source control can be achieved by resection or restoration of the infectious or perforated visceral organ depending on the etiology and localization, on the extent of the peritoneal contamination, and on pre-existing comorbidities of the patient [1, 5, 6]. Dilution of the bacterial load by peritoneal lavage using saline fluids, antibiotic or antiseptic suspensions is often performed. However, none of these solutions have a proven positive effect on the outcome of secondary peritonitis [5]. It can even wash out or damage mesothelial cells, which play an important role in the patients’ immune responses [7]. The old saying “the solution to pollution is dilution” should be abandoned regarding the peritoneal cavity and can even be harmful.

### Resuscitation

Secondary peritonitis, and possible subsequent sepsis, dictates the need for adequate resuscitation following the Surviving Sepsis Campaign Guidelines. Sepsis can lead to MOF due to inadequate tissue perfusion. Resuscitation encompasses all measures to maintain or enhance organ perfusion and oxygenation. Adequate resuscitation within 6 h of the onset of sepsis increases survival [8].

### Antimicrobial therapy

Early administration of empiric antibiotic regimens is of utmost importance. Every 30-min delay in administering antibiotics after diagnosing secondary peritonitis increases death rates with an odds ratio of 1.021 (95 % CI: 1.003–1.038) [9]. The benefit of early adequate antibiotic coverage is demonstrated by the reduction of mortality in patients with bacteremia admitted to the ICU (risk reduction 33 %) [10]. A Cochrane review on this subject describes the comparable effectiveness of available regimens [11]. However, one needs to adjust the regimen of choice depending on the expected microbes; regimens can be adjusted when culture results become available [12].

### Antifungal therapy

A considerable proportion of peritonitis patients are admitted to the ICU where colonization with yeasts and fungal strains, mainly Candida, is common [13]. A meta-analysis has shown that the risk of yeast infections is reduced by both single-drug antifungal prophylaxis (SAP) and selective bowel decontamination [SBD; OR: 0.54 (95 % CI: 0.39–0.75; NNT 20) and 0.29 (95 % CI: 0.18–0.45; NNT: 18), respectively]. Also death due to yeast infections is reduced after prophylaxis, irrespective of SAP or SBD (combined OR: 0.23; 95 % CI: 0.09−0.6; NNT: 41) [14]. Because of the increasing number of yeast infections prophylaxis is advised for high-risk patients. Known risk factors are surgery, nosocomial peritonitis, high digestive tract perforation, immune deficiency, long-term antibiotic use, acute renal failure, and a central venous access [15].

## Treatment following emergency laparotomy

### Surgical strategy

Different surgical strategies are followed for mild peritonitis and severe peritonitis. It is important to realize, however, that to date there is no strict consensus in the literature on the definition of severe peritonitis, on which clinical score to use, and on what cut-off value adequately distinguishes the various degrees of severity of peritonitis. The use of the Acute Physiology and Chronic Health Evaluation (APACHE) II score is most accepted, with mild peritonitis defined as an APACHE II score of ≤ 10, and severe peritonitis as a score of > 10 [3]. In mild peritonitis clinical deterioration or lack of improvement within the first postoperative period following the emergency laparotomy dictates the necessity for a relaparotomy, referred to as the “on-demand” strategy [3]. Severe peritonitis used to be addressed by more aggressive surgical approaches such as radical peritoneal debridement, “open abdomen” (OA) treatment, and planned relaparotomy strategy. Both radical debridement and OA strategy were discarded after research showed higher morbidity and mortality rates [16]. Notwithstanding the negative results with planned OA, in recent years a trauma principle termed “damage control surgery” has gained popularity in peritonitis settings. Here, hit-and-run surgery is performed for acute severe peritonitis, the OA is temporary closed with a mesh inlay of negative pressure wound therapy on the OA, and a commitment for delayed abdominal closure is made but not always achieved. This strategy involves multiple sessions of abdominal surgery, spread over several days, even weeks. The clinical outcome of such damage control surgery is largely unknown as only small retrospective case series have been published, as recently reviewed [17].

**Fig. 1:**
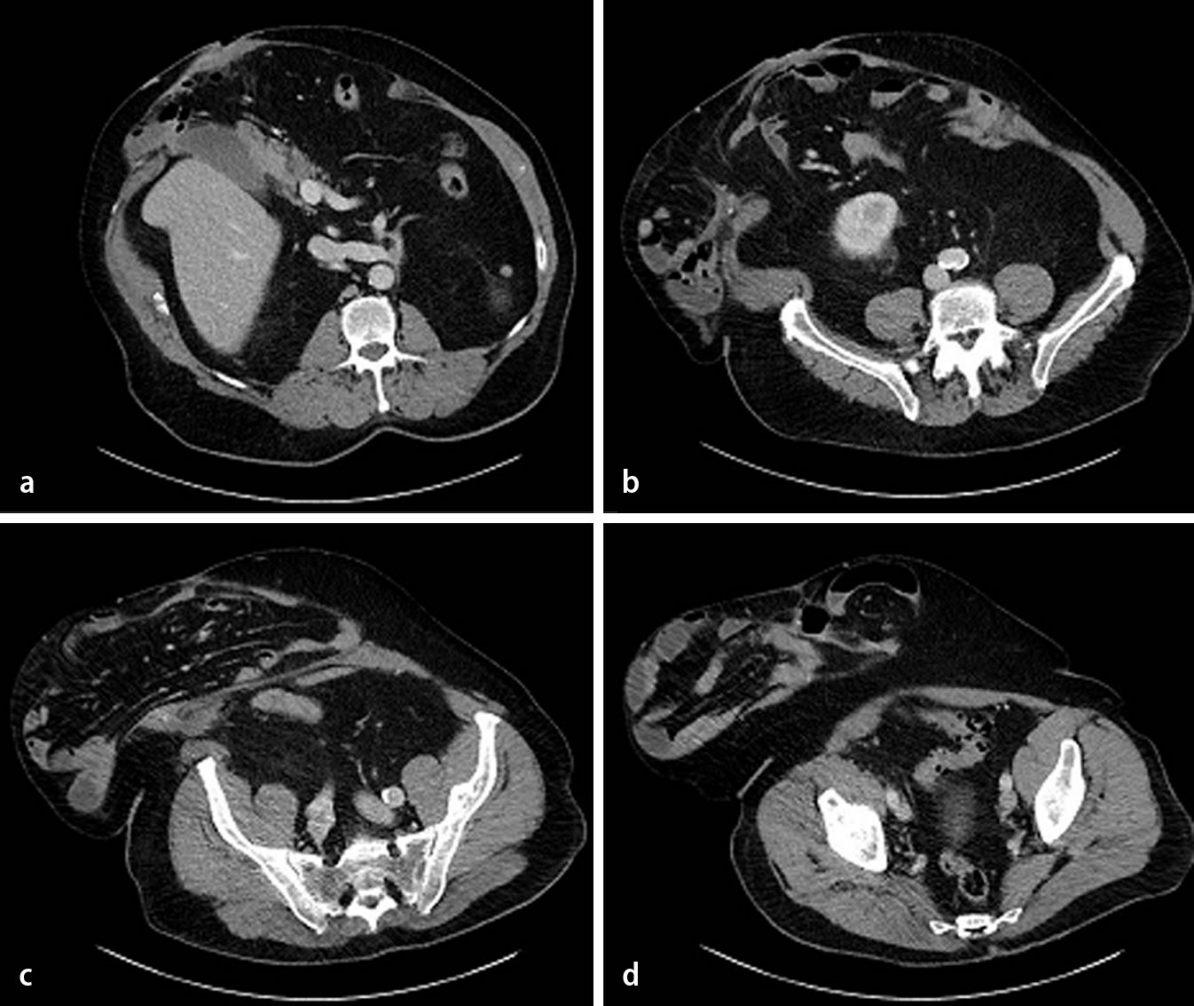
**a–d ** The catastrophic consequences of a planned open abdomen: fistula in an open abdomen and remnants of synthetic mesh used for temporary closure

OA gives direct access to the abdomen for relaparotomy and is thought to prevent abdominal compartment syndrome. However, known complications of OA are anastomotic leakage, the development of enteroatmospheric fistula (10–20 %), ileus, excessive fluid loss, bleeding from the OA surface, secondary infection rates of > 80 %, residual fascial dehiscence (ventral hernia), and increased mortality rates. Also, multiple techniques are adhered to. The overall quality of evidence is poor and true recommendations cannot be made [18–20]. Considering all the substantial negative effects of OA our recommendation is always to close the abdomen where possible, and not opt for a planned OA. If owing to visceral edema the abdomen cannot be closed, various temporary closure devices are available [20]. Delayed fascial closure is not always achieved, enteric fistula rates remain significant, and the most widely applied closure techniques require multiple dressing changes and OR visits (Fig. 1; [20]). A potential alternative abdominal closure technique is the use of a biologic mesh. Early closure of the abdomen during the initial operation or shortly thereafter in these contaminated fields with a non-cross-linked biologic mesh may provide an immediate solution and can theoretically reduce the rate of fistula formation and hernia recurrence.

Planned relaparotomy strategy means a relaparotomy is performed every 2–3 days until the abdominal cavity is macroscopically free from infection (“clean”) regardless of the patient’s clinical conditions [5, 21]. A planned strategy was thought to have the advantage of allowing early identification and treatment of persistent peritonitis or new infective foci, but in fact it increases the number of unnecessary relaparotomies [3]. Outcome data indicate that for severe peritonitis, too, the on-demand surgical strategy is the treatment of choice, rather than the planned relaparotomy strategy [3]. There is even evidence that multiple relaparotomies actually increase the systemic inflammatory mediator response resulting in an increased incidence of MOF and mortality [22].

**Fig. 2:**
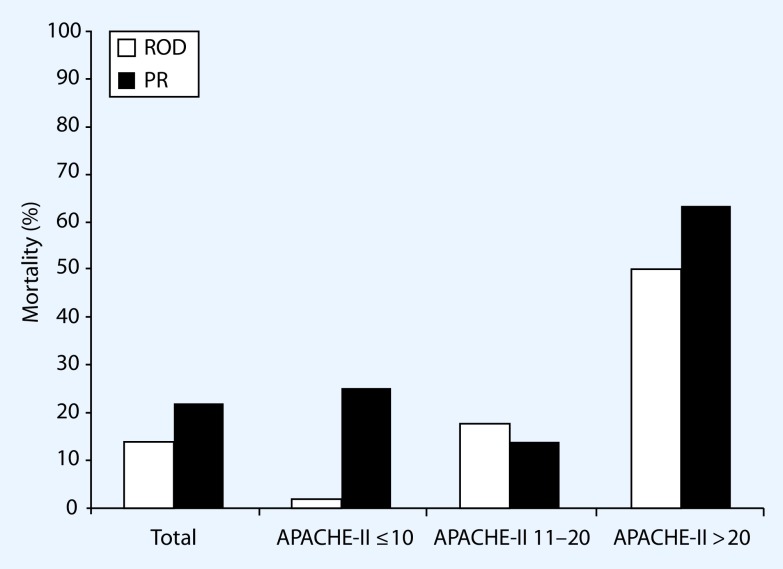
Mortality rates stratified for relaparotomy on demand (□; *ROD*) and planned relaparotomy (■; *PR*) with severity of disease for patients included in the RELAP trial

**Fig. 3:**
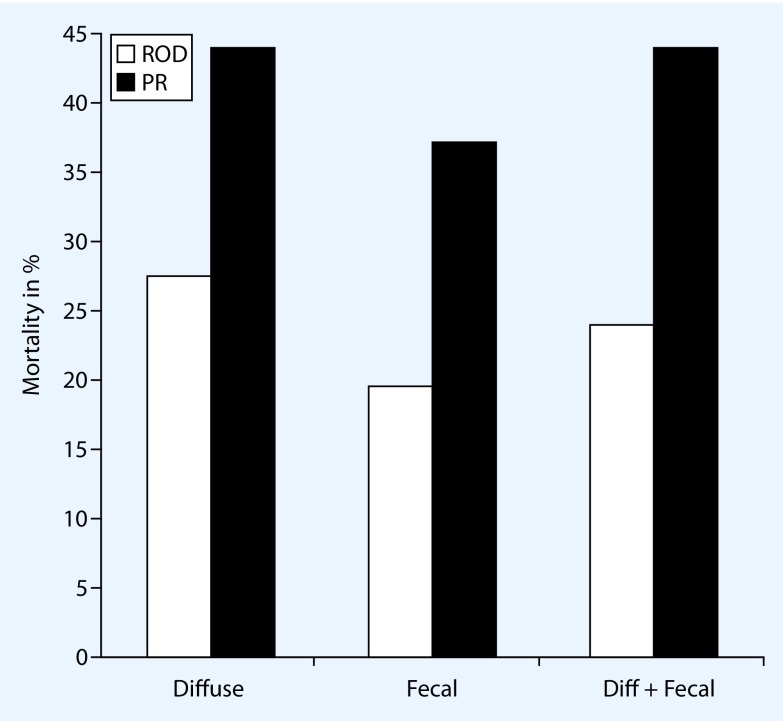
Mortality rates stratified for relaparotomy on demand (□; *ROD*) and planned relaparotomy (■; *PR*) with type of contamination for patients included in the RELAP trial

Our study group performed a randomized controlled trial comparing planned relaparotomy with on-demand relaparotomy strategy (RELAP trial) [3]. In total, 232 patients with moderate to severe secondary peritonitis (APACHE II score > 10) were included with 116 patients treated in each strategy arm. Mortality was 29 % in the on-demand group versus 36 % in the planned relaparotomy group (*p* = 0.22). Also for severely ill patients with secondary peritonitis (APACHE II score > 20; Fig. 2), the mortality outcome was not in favor of planned relaparotomy. This finding opposes the widely accepted theory that especially ill patients in particular benefit from planned relaparotomy. Another unconfirmed dogma is that planned relaparotomy is imperative in the case of fecal contamination at initial laparotomy. Mortality rates are higher for planned relaparotomy than for on-demand relaparotomy in diffuse purulent or fecal peritonitis (Fig. 3; [3, 16]). An on-demand strategy safely reduces health-care needs owing to significantly shorter ICU and hospital stays. This reduction of care utility saves up to approximately € 17,500 per patient on medical costs [23]. Patients treated with the on-demand approach received fewer relaparotomies (113 vs. 233 in the planned group); 58 % of patients treated by on-demand relaparotomy never needed a relaparotomy. Furthermore, the percentage of negative relaparotomies (no persistent or new infectious focus) was lower in the on-demand group (31 vs. 66%) [3]. A negative relaparotomy can be considered as unnecessary and even hazardous for the patient. Improving patient selection for intervention by computed tomography (CT) imaging can theoretically further reduce the proportion of negative relaparotomies in the on-demand strategy. Moreover, emphasis on percutaneous drainage of infected fluid collections also can reduce the need for relaparotomy.

Despite the positive clinical findings for relaparotomy on demand in this large randomized trial, the on-demand strategy being described as “the conventional treatment strategy” in other research articles, and the beneficial economic impact, planned relaparotomies are still performed today. Introduction of the damage control strategy for peritonitis patients is a threat to the beneficial effects of on-demand relaparotomy.

### Monitoring

There is no decisional aid to support timely patient selection, and the decision for relaparotomy is based on subjective interpretation of undefined variables. There are no existing prediction scores that are apt or validated to predict ongoing peritonitis [24]. Early postoperative clinical variables seem most predictive for ongoing sepsis [25]. Hence, intensive monitoring in the direct postoperative setting is essential to be able to reconsider the need of a relaparotomy every 24 h.

Research on specific immunologic markers predicting abdominal sepsis is sporadic. Gans et al. have recently published a meta-analysis on the predictive value of C-reactive protein (CRP) to rule out infectious complications following major abdominal surgery. They conclude that infectious complications after major abdominal surgery are very unlikely in patients with a CRP below 159 mg/l on the third postoperative day [26]. Another study, specifically on anastomotic leakage, has also shown a negative predictive value of CRP on postoperative days 3, 4, and 5, but a low positive predictive value [27]. Others have found CRP and leukocyte counts to be of low additional value for predicting anastomotic leakage, e.g., following laparoscopic colorectal resections [28].

Interleukin (IL)-6 seems to be a promising early marker of overall and postoperative complications and sepsis following elective major abdominal surgery, distinguishing patients at risk as early as on the first postoperative day whereas CRP starts to distinguish from day 3 onward [29]. Another possible marker is preoperative measured intestinal fatty acid-binding protein, which has additional value in the assessment of risk of anastomotic leakage. A combination of CRP with calprotectin also showed high diagnostic accuracy for anastomotic leakage [30]. Unfortunately, data on the added value of these markers in selecting patients with ongoing peritonitis in often already septic patients are not available. Implementation of these markers in close monitoring of patients for ongoing abdominal sepsis definitely deserves additional investigation.

**Fig. 4:**
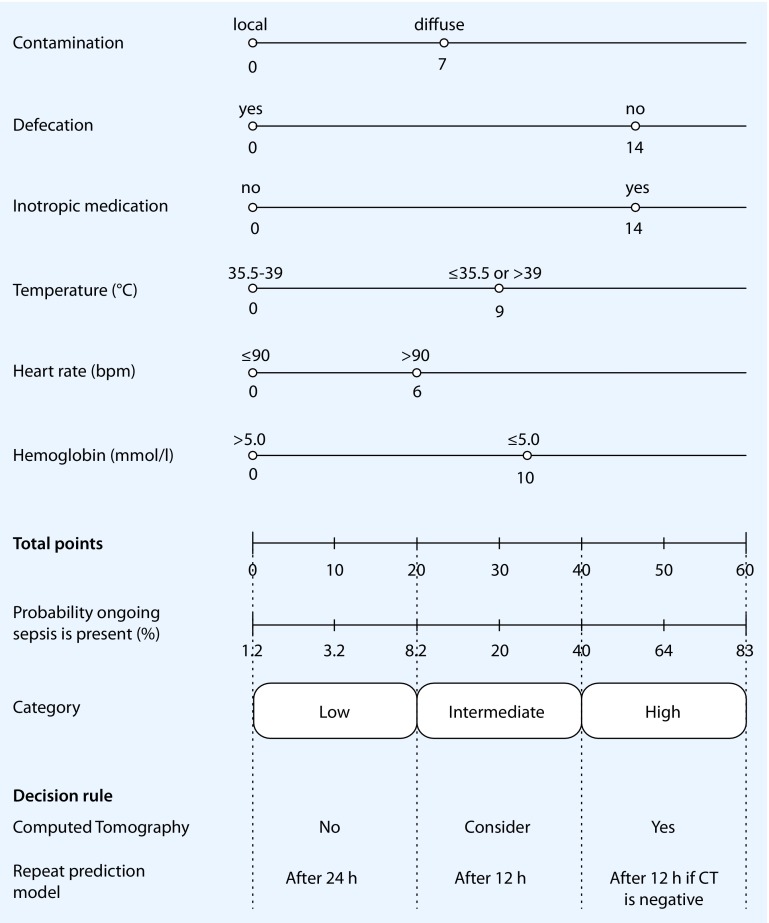
Nomogram depicting the decision tool for predicting ongoing abdominal sepsis with advice regarding monitoring and performing imaging studies [25]

Another problem in the monitoring and selection of patients for relaparotomy is the unknown true value of CT for (ongoing) abdominal infection in an early postoperative setting. The positive predictive value of CT for abdominal sepsis following elective abdominal surgery is 0.71 (95 % CI: 0.57–0.83), hence leaving an important margin of insecurity. However, the negative predictive value is 0.15 (95 % CI: 0.06–0.32), thus quite reliable [31]. In the RELAP trial, CT had been performed in only 18 % of relaparotomy on demand patients during the first week following initial laparotomy, even though most of the relaparotomies were performed during this time span. The use of CT in selecting patients for relaparotomy and subsequent knowledge on the interpretation of early postoperative findings will probably enhance the efficacy of the relaparotomy on demand strategy [25, 31]. We have developed a decision tool (Fig. 4) to determine the probability of ongoing sepsis from an abdominal infectious focus in patients operated on for secondary peritonitis, which is based on early postoperative predictive factors and can be used every 12–24 h. Based on the risk category, a CT or prompt reassessment of the prediction model is advised [25]. This decision tool was recently externally validated (Atema et al., manuscript submitted). A total of 161 assessments using the decision tool were performed for 69 patients. The discriminative capacity of the decision tool score was fair (area under the receiving operator curve of 0.79). The incidence rate of ongoing sepsis differed significantly between three score categories. The negative predictive value of a decision tool score categorized as “low” was 89%. In clinical practice this negative predictive value can aid postoperative decision making.

### Optimization of multidisciplinary care

The treatment of secondary peritonitis demands a multidisciplinary approach with the surgeon, intensivist, radiologist, and microbiologist working together very closely. Approximately 40 % of all patients diagnosed with secondary peritonitis will need ICU treatment. To date there is concern about the influence of ICU variables influencing mortality and morbidity. It is known from Dutch studies that a higher treatment volume ICU reduces overall mortality in patients with severe sepsis [32]. Centralization of care for patients with secondary peritonitis is neither workable nor possible, considering the high incidence. However, one should consider referring critically ill patients to a high-level ICU with a closed format and a 24/7 availability of intensivists and intervention radiologists.

## Conclusion

The treatment of secondary peritonitis comprises multiple aspects. Improving only one aspect will not lead to a drastic reduction in mortality and morbidity. The multidisciplinary approach as well as the diagnostic and decisional processes need to be improved. Examples of important advances in peritonitis treatment are preemptive antifungal therapy in high-risk patients, increasing doubt about the benefits of abdominal lavage, acknowledgement of the importance of closing the abdomen, and applying the relaparotomy on demand strategy to all peritonitis patients regardless of disease severity. We stress the importance of close monitoring of peritonitis patients, intensive use of diagnostics, and 24/7 decision making. Planned relaparotomy eases the doctor’s mind, but it interferes with our patients’ well-being.
